# Acute Limb Ischemia in COVID-19 Disease: A Mysterious Coagulopathy

**DOI:** 10.7759/cureus.9167

**Published:** 2020-07-13

**Authors:** Shamsuddin Anwar, Sudeep Acharya, Sohaib Shabih, Anthony Khabut

**Affiliations:** 1 Internal Medicine, Staten Island University Hospital, Northwell Health, Staten Island, USA

**Keywords:** covid 19, limb ischemia, hematology-oncology, world pandemic

## Abstract

Severe coronavirus disease 2019 (COVID-19) as a respiratory tract infection has been noted to be a causative agent for acute respiratory distress syndrome, shock, and multiple organ failure. It is also being suggested that COVID-19 results in serious systemic coagulopathies similar to disseminated intravascular coagulation. We describe a case of severe arterial thrombosis induced by COVID-19 infection along with its pathological implications.

## Introduction

Coronavirus disease 2019 (COVID-19) has been declared a worldwide pandemic and is giving rise to a myriad of coagulation abnormalities along with the constitutional and respiratory tract symptoms [1]. The most common coagulation abnormalities noted so far include thrombotic events in major venous vessels, with various derangements in inflammatory markers especially D-dimers and fibrinogen [2]. In this case report, we describe an interesting clinical scenario of arterial thrombus formation in a COVID-19 patient despite being on full anti-coagulation, resulting in limb-threatening limb ischemia.

## Case presentation

A 58-year-old male with a pertinent past medical history of dyslipidemia initially presented to the hospital due to acute shortness of breath and subjective fever for the past week. On presentation, he was swabbed for novel coronavirus, which resulted as positive. For the first five days, he was monitored on the regular medical floor for his oxygen requirements, which fluctuated between 2 and 3 liters on the nasal cannula. He was initiated on hydroxychloroquine and azithromycin for five days, and his progression of inflammatory markers was trending every 48 hours (Table 1).

**Table 1:** Inflammatory markers on admission

Inflammatory Markers	Results
C-reactive protein	25.23
Lactose dehydrogenase	558
D-dimers	15,653
Procalcitonin	0.17
Ferritin	1,311
Fibrinogen	312

**Figure 1:**
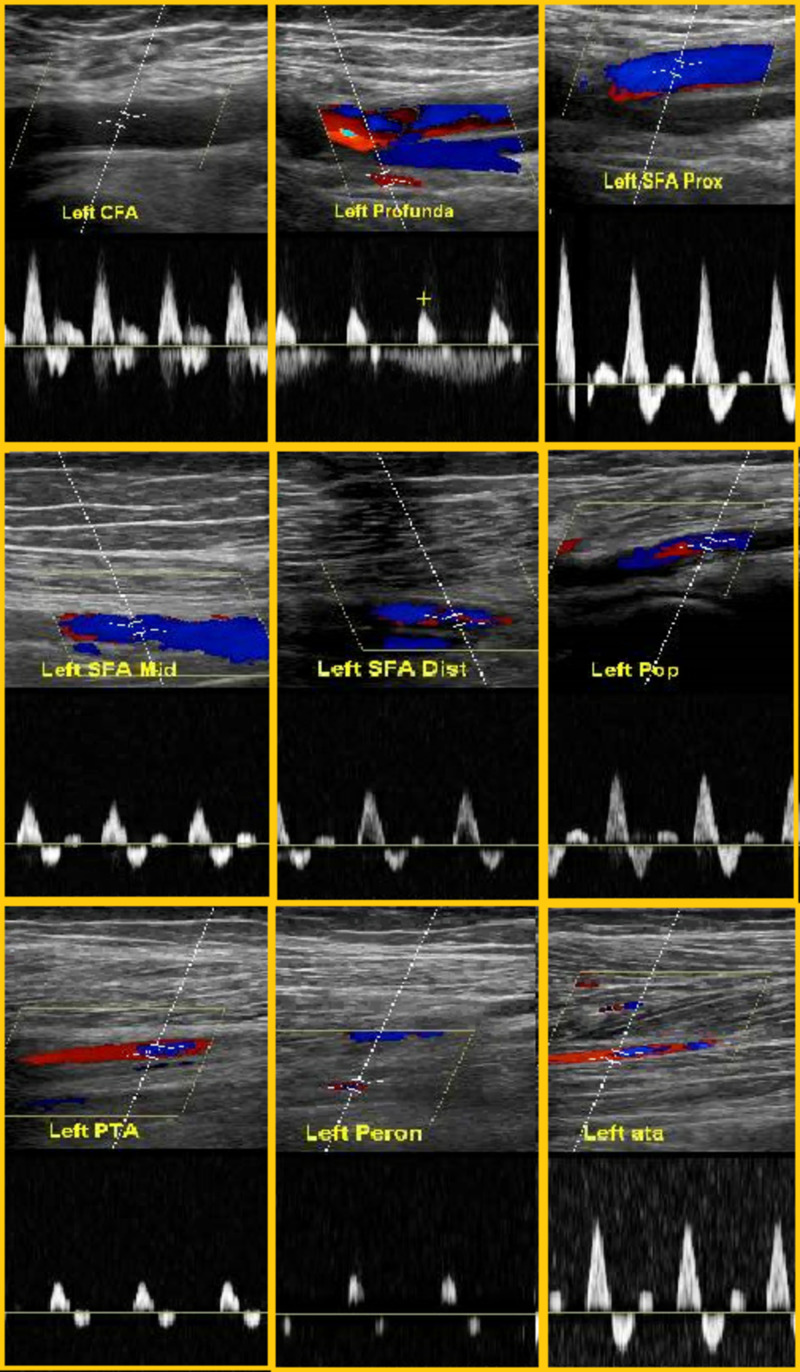
Arterial ultrasound of the left lower extremity with waveforms The arterial ultrasound of the left lower extremity did not show any occlusions. CFA, common femoral artery; SFA Prox, superficial femoral artery proximal; SFA Mid, superficial femoral artery mid; SFA Dis, superficial femoral artery distal; Pop, popliteal; PTA, posterior tibial artery; Peron, peroneal; ATA, anterior tibial artery

On the fifth day of admission, the patient’s oxygen requirement worsened to the point that he had to be transitioned to a non-rebreather mask on 15 liters. His other vital signs included a blood pressure of 130/75 mm Hg, respiratory rate of 22 breaths per minute, temperature of 99.4 degrees Celsius, and pulse oxygenation ranging from 90% to 94%. He was transferred to the critical care unit for closer monitoring. He was initiated on intravenous corticosteroids and interleukin (IL) 1 inhibitor anakinra. On the seventh day, he started complaining about paleness and cold left lower extremity as compared to the right side. On examination, there were no motor or sensory deficits; however, pulses were detectable only on Doppler ultrasound (Figure 1). Urgent vascular surgery evaluation was conducted, and the bilateral arterial duplex was performed, which demonstrated normal arterial flow. The patient, however, was switched to therapeutic low molecular weight heparin (LMWH) given the high suspicion for prothrombotic state associated with cytokine release syndrome.

**Figure 2:**
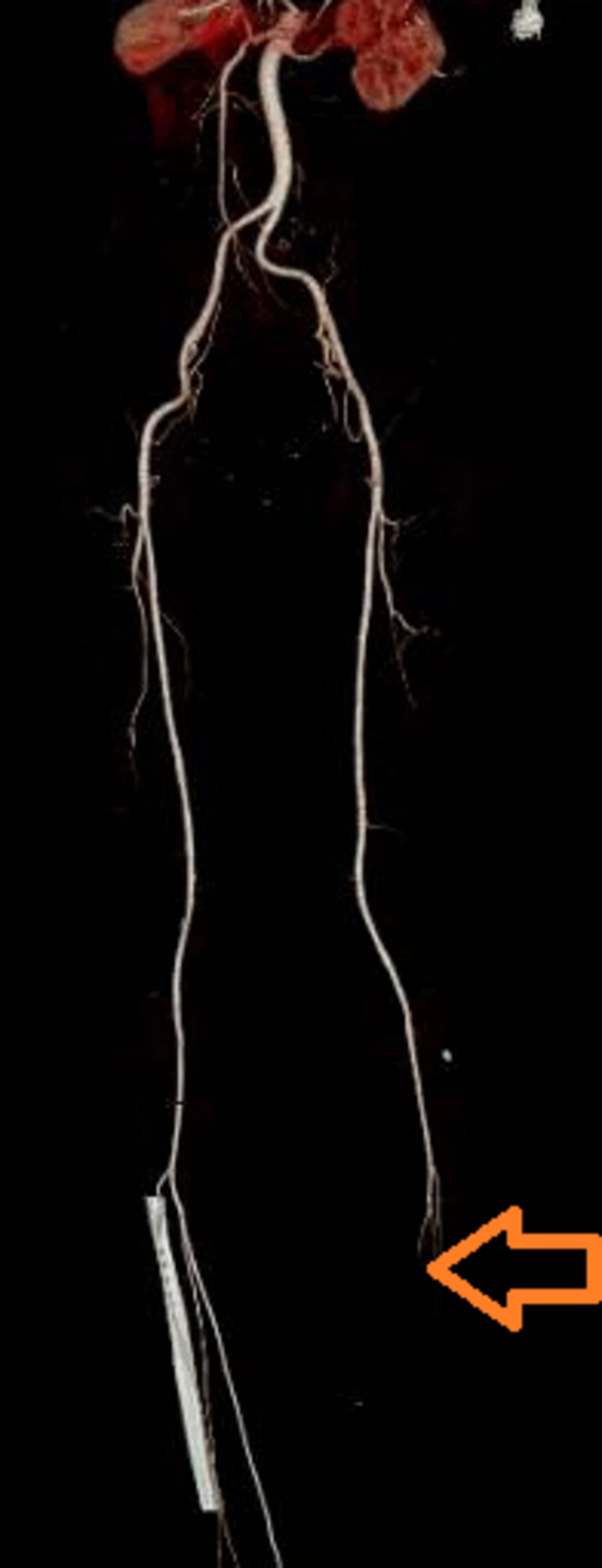
CT angiogram of the lower extremities The arrowhead showing reduced blood flow with blockage in the arterial vessels of the left lower extremity.

His oxygen requirements were successfully titrated down to 6 liters by the 10th day of hospitalization; however, he continued to complain about his left cold foot, which gradually progressed to develop cyanosis. The patient at this time was switched to intravenous heparin drip in consideration of unstable peripheral vascular disease. With no improvement noted in his cyanosis, CT angiogram was obtained, which demonstrated filling defects in the main left femoral artery suspicious of thrombus (Figure 2). Decreased blood flow was also noted in the left popliteal, posterior tibial, peroneal, and anterior tibial arteries.

Heparin drip was continued and aspirin 81 milligrams was added in the regimen. Despite being on full anticoagulation, the patient was developing arterial thromboses, which prompted hematology evaluation for hypercoagulable screening. Peripheral smear was also performed, which did not reveal any schistocytes; the red blood cells, leukocytes, and platelets demonstrated normal features.

Extensive blood workup including genetic studies was obtained, which are summarized in Table 2. The mildly elevated anticardiolipin immunoglobulin M was considered secondary to inflammation and deemed non-specific. Ultimately, vascular surgery was performed, with angioplasty as limb salvage therapy in the femoral, popliteal, and tibial arteries. The re-vascularization was followed with below-the-ankle amputation of the necrotic dead tissue.

**Table 2:** Gene mutation and markers for hypercoagulability profile

Tests and Markers	Results
Beta 2 glycoprotein	Negative
Anticardiolipin immunoglobulin G	Negative
Anticardiolipin immunoglobulin M	Slightly positive
Anti-nuclear and anti-double-stranded DNA antibodies	Negative
JAK 2-V617 mutation	Negative
CALR gene mutation	Negative
Flow cytometry	Normal
MPL gene mutation	Negative
JAK 2 exon mutation	Negative
Erythropoietin	Normal

## Discussion

The inflammatory process has been described to be closely interlinked with and resulting in a pro-thrombotic state in humans in several studies. When the inflammatory process damages the endothelial linings of the macro- and microvasculature, it disrupts the normal antithrombotic and anti-inflammatory mechanisms, resulting in unchecked activation of the thrombotic cascade. [1] COVID-19 was declared a worldwide pandemic by the World Health Organization on March 11, 2020. While many patients develop mild-to-moderate symptoms (fever, fatigue, myalgia, headache, diarrhea, dry cough, and dyspnea), several patients have been described to have severe systemic disease, resulting in a myriad of coagulation abnormalities [2].

The suggested mechanism through which SARS-CoV-2 (severe acute respiratory syndrome coronavirus 2) causes the systemic disease is through angiotensin-converting enzyme 2 (ACE-2) receptors. The SARS-CoV-2 proteins infect human cells through ACE-2 receptors, which are expressed in various degrees in alveolar epithelial cells, large and small arterial endothelial cells, small intestinal epithelial cells, immune tissues, and various other types of cells [3]. SARS-CoV-2 directly attacks vascular endothelial cells and activates the coagulation cascade after causing endothelial injury. This pathologic insult is suggested to result in excessive cytokine release and storm from activating of widespread coagulation factors while inhibiting fibrinolysis causing extensive thrombosis similar to disseminated intravascular coagulation. IL-6 is a key factor in SARS-CoV-2 induced inflammatory storm. While IL-6 can stimulate the liver to synthesize fibrinogen and thrombopoietin, it also upregulates the expression of vascular endothelial growth factor to disrupt the stability of vascular barrier and stimulate monocytes to express more tissue factors, thereby activating the extrinsic pathway of coagulation [4]. These coagulation abnormalities along with elevated D-dimers are likely indicators for higher mortality predisposing the patients to a variety of ischemic and thrombotic events [5].

Various elevated markers including D-dimers, partial thromboplastin time (PTT), prothrombin time (PT), fibrinogen, fibrin degradation products (FDP), and IL- 6 have been described to determine the progression of sepsis-induced prothrombotic disease secondary to SARS-CoV-2 [6]. It is interesting to note that although several studies have shown severe thrombotic events associated with SARS-Cov-2 sepsis, limited data are available to suggest uncontrolled bleeding diathesis caused by COVID-19 except for intracerebral hemorrhages [7]. A small retrospective analysis of 20 critically ill COVID-19 patients was conducted who had different degrees of limb ischemia and variable re-vascularization success [8]. Our clinical scenario was unique in describing the development of acute limb ischemia despite being on full therapeutic anticoagulation with LMWH. Although the initial arterial Doppler ultrasound of the lower extremities failed to show any thrombosis, our patient was initiated on 1 mg/kg enoxaparin twice daily. With no significant improvement noted, a CT angiogram was performed, which demonstrated several filling defects.

As not enough data are available to confirm COVID-19 as being the culprit for arterial thrombosis, it mandated a hematology-oncology evaluation to rule out rare causes of thrombosis including malignancy. Extensive workup including gene mutations and flow cytometry was performed and resulted in negative. The patient finally underwent limb salvage therapy through angioplasty with below-the-ankle amputation of the gangrenous tissue [9,10].

The role of anticoagulants in preventing thrombotic complications and improving the overall prognosis in COVID-19 has not been clearly established. Tang et al. in their study of 499 patients with COVID-19, out of which 99 patients received LMWH therapy, reported that anticoagulant therapy mainly with LMWH appears to be associated with better outcomes in severe COVID-19 patients [11]. It was interesting to note that despite being on full anticoagulation with LMWH and later with unfractionated heparin, our patient’s gangrene continued to worsen, ultimately requiring vascular intervention.

There is supporting evidence available for using prophylactic anticoagulation for venous thromboembolism; however, it is unclear whether prophylactic or full anticoagulation will be beneficial for arterial thrombosis caused by COVID-19 [12]. In current medical practice, the patients with viable tissue and marginally threatened limb undergo vascular intervention for arterial thrombolysis and thrombectomy. Limbs with irreversible ischemia require amputation, as in our clinical case.

## Conclusions

The subject of SARS-CoV-2 induced coagulation abnormalities leading to thrombotic complications requires further large-scale studies thoroughly at the molecular level. For critically ill patients with COVID-19, special attention should be paid to abnormal coagulation dysfunction and microcirculatory disorders. Prophylactic anticoagulation has been reported to be beneficial for venous thromboembolism induced by COVID-19; however, arterial thromboembolic events as seen in our patient would require prompt vascularization due to anticoagulation failure.
